# New Small Molecule Drugs for Thrombocytopenia: Chemical, Pharmacological, and Therapeutic Use Considerations

**DOI:** 10.3390/ijms20123013

**Published:** 2019-06-20

**Authors:** Page Clemons Bankston, Rami A. Al-Horani

**Affiliations:** Division of Basic Pharmaceutical Sciences, College of Pharmacy, Xavier University of Louisiana, New Orleans, LA 70125, USA; pclemons@xula.edu

**Keywords:** lusutrombopag, avatrombopag, fostamatinib

## Abstract

This review provides details about three small molecules that were recently approved by the FDA for the treatment of thrombocytopenia. The new treatments include lusutrombopag, avatrombopag, and fostamatinib. The first two drugs are orally active thrombopoietin receptor (TPO-R) agonists which are FDA-approved for the treatment of thrombocytopenia in adult patients with chronic liver disease who are scheduled to undergo a procedure. Fostamatinib is orally active prodrug that, after activation, becomes spleen tyrosine kinase (SYK) inhibitor. Fostamatinib is currently used to treat chronic and refractory immune thrombocytopenia in patients who have had insufficient response to previous treatment. Chemical structures, available dosage forms, recommended dosing, pharmacokinetics, results of toxicity studies in animals, most frequent adverse effects, significant outcomes of the corresponding clinical trials, and their use in specific patient populations are thoroughly described. Described also is a comparative summary of the different aspects of five currently available therapies targeting TPO-R or SYK for the treatment of thrombocytopenia.

## 1. Introduction

Thrombocytopenia is a pathological condition in which the number of platelets falls below the normal range. A normal platelet count in adults is within the range of 150–450 × 10^3^ platelets/µL of blood. Platelets, also known as thrombocytes, are small blood cells that are produced by bone marrow along with the red and white blood cells and have a life span of 8–10 days. Platelets are essential for hemostasis, a mechanism by which physiological blood clotting takes place to stop bleeding at the site of blood vessel injury. In this process, platelets are activated by one or more factors including thrombin, adenosine diphosphate, thromboxane A2, or collagen, and subsequently, adhere and aggregate at the site of injury. The aggregated platelets along with fibrin and red blood cells form the blood clot. Therefore, when the platelet count falls below about 50 × 10^3^ cells/µL, the blood loses its ability to adequately clot and bleeding may occur even after minor injury. Nevertheless, the most significant risk of bleeding largely occurs when the platelet count is below 10–20 × 10^3^ platelets/µL of blood. At these levels, bleeding may happen without serious injury [1].

Problems in platelets production, distribution, or destruction lead to thrombocytopenia [1]. In fact, many factors can cause thrombocytopenia, and these factors can be inherited or acquired. Decreased production can be attributed to leukemia and cancer chemotherapy-induced myelosuppression, aplastic anemia, viral infections as in the case of human immunodeficiency viral infection, hepatitis C viral infection, or Epstein–Barr viral infection, and heavy alcohol consumption. Furthermore, platelets may become entrapped in a distended spleen as in the case of liver cirrhosis, Gaucher disease, and myelofibrosis which subsequently reduces the platelet count in the blood stream. Huge red blood cell transfusions may also decrease the platelet count. Platelets destruction could be attributed to multiple reasons including pregnancy and bacteremia [2,3]. Autoimmune diseases can also destroy the body’s platelets resulting in either primary or secondary immune thrombocytopenia. In primary immune thrombocytopenia, immune dysregulation of unknown etiology results in the formation of auto-antibodies or immune complexes that increase the peripheral destruction of platelets by binding to platelets and causing platelet phagocytosis, accompanied by T-cell and potentially complement-facilitated lysis. The production of new platelets is also blocked because the antibodies bind to megakaryocytes in the bone marrow, and subsequently, decrease the megakaryocytes number and inhibit their maturation. On the contrary, preexisting conditions such as lupus and rheumatoid arthritis, which are autoimmune diseases, may lead to secondary immune thrombocytopenia. Other serious conditions that can cause secondary immune thrombocytopenia include thrombotic thrombocytopenic purpura, disseminated intravascular coagulation, and hemolytic uremic syndrome [1]. Lastly, thrombocytopenia can also be triggered by the use of drugs, some of which trigger immune other trigger nonimmune thrombocytopenia (Table 1) [4,5].

Treatment for thrombocytopenia depends on its underlying cause and severity. In mild conditions, a treatment may not be needed, however in severe conditions, blood or platelet transfusions may be needed. Thrombocytopenia often improves when its underlying cause is treated. For instance, if thrombocytopenia was induced by a drug, most patients recover after stopping the drug. For heparin-induced thrombocytopenia (HIT), stopping heparin is not sufficient and another anticoagulant is needed (fondaparinux, argatroban, or bivalirudin) to prevent potential blood clotting [6,7]. In the case of immune thrombocytopenia, the most common treatments include corticosteroids which decrease the production of antibodies against platelets, and subsequently, increase the platelet count within 2–4 weeks. Intravenous gamma globulin can also be used to slow the rate of platelet destruction. Although intravenous gamma globulin therapy works faster than steroids, yet its effect typically lasts several days to a couple weeks. The monoclonal antibody rituximab or some of the recently approved small molecule drugs can be used if the initial treatments were not adequate. Splenectomy is endorsed for patients who do not benefit from any of the above treatments for immune thrombocytopenia [8,9].

This review will focus on the chemical, pharmacological, and therapeutic use aspects of the recently approved small molecule drugs for thrombocytopenia which include lusutrombopag, avatrombopag, and fostamatinib.

## 2. The Development of Small Molecule Drugs for Thrombocytopenia

### 2.1. Thrombopoietin Receptor (TPO-R)

Thrombopoietin (TPO) is the endogenous regulator of platelet production. TPO is produced primarily in the liver and binds to and activates a specific thrombopoietin receptor (TPO-R) on the membrane of platelets, megakaryocytes, hemangioblasts, and hematopoietic stem cells [10,11]. Therefore, the receptor is important for the regulation of platelet production as well as the maintenance of hematopoietic stem cells. The receptor was first identified in 1992. It contains 635 amino acids that are organized in three structural domains: The extracellular domain for cytokine binding, the transmembrane domain, and the cytoplasmic domain which binds signaling molecules [10,11]. The endogenous ligand for TPO-R (thrombopoietin) binds to the extracellular domain of partially pre-dimerized cell surface receptor. This binding is thought to bring about a change in the monomer–dimer equilibrium and/or in the receptor dimer arrangement, and thus, initiates a series of signaling events in the target cell. Importantly, TPO-R lacks intrinsic kinase activity, yet it exploits the Janus kinase (JAK) protein family to transduce a signal from the extracellular cytokine to the nucleus in the target cell. Particularly, TYK2 and JAK2 are associated with the cytoplasmic domain of TPO-R and get activated by phosphorylation upon signaling. Subsequently, the activated JAKs phosphorylate the receptor and the signal transducer and activator of transcription (STAT) 1, 3, and 5, and activate mitogen-activated protein kinase (MAPK) and phosphatidylinositol-3 kinase (PI3K) pathways [12]. Studies suggest that the lack of functional TPO-R in human leads to congenital amegakaryocytic thrombocytopenia, a rare condition in which infants develop platelets and megakaryocytes deficiency that results in multilineage failure [13,14,15].

**Figure 1:**
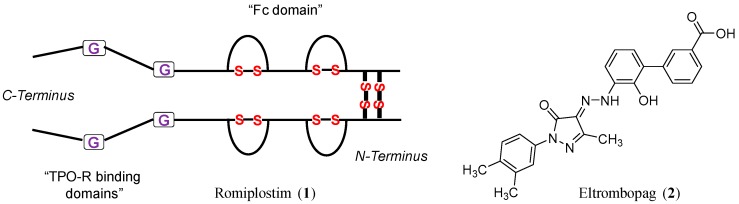
Structural representations of romiplostim (**1**) and eltrombopag (**2**). The representation of romiplostim shows the TPO-R binding domain at the C-terminus and the Fc domain at the N-terminus. Glycine bridges link the two domains. Inter- and intra-disulfide bonds are also shown. The structure of eltrombopag shows the biphenyl carboxylic acid domain “right-hand domain” and the dimethylphenyl domain “left-hand domain” both of which are linked together by dihydro-pyrazol-4-ylidene-hydrazino bridge.

The discovery of TPO as the endogenous ligand for TPO-R has motivated the scientific community to find ways to use it as a platelet production stimulator. The 1^st^ generation of TPO-R agonists used in clinical trials were recombinant human TPO and PEGylated TPO-derived peptide [16,17,18,19]. The two forms increased thrombopoiesis in both healthy and thrombocytopenic subjects. Yet, the two trials were terminated and the development of the two treatments was stopped because some healthy subjects developed thrombocytopenia due to the formation of antibodies that cross-reacted with endogenous TPO. Thus, the development of 2^nd^ generation thrombopoietic agents has focused on minimizing structural similarities with TPO. The 2^nd^ generation TPO-R agonists are classified into three types including (1) TPO peptide mimetics; (2) TPO non-peptide mimetics; and (3) TPO-R antibodies. Previously, two receptor agonists were approved by the FDA: romiplostim (**1**) and eltrombopag (**2**) (Figure 1) [16,17,18,19]. In one hand, romiplostim is 14-amino acid peptide with no homology to TPO, yet with high affinity for TPO-R [20,21,22,23]. In the other hand, eltrombopag is allosteric small molecule that targets the extracellular domain of TPO-R and causes a conformational change in the transmembrane and intracellular domains of the receptors allowing physiological activation through receptor dimerization [24,25,26,27]. Romiplostim and eltrombopag are approved for the treatment of chronic immune thrombocytopenia and thrombocytopenia of liver diseases. In 2018, two more small molecule agonists of TPO-R were approved. Lusutrombopag and avatrombopag are orally bioavailable drugs that interact with the transmembrane portion of TPO-Rs on megakaryocytes, and subsequently, stimulate the proliferation and differentiation of megakaryocytes from bone marrow progenitor cells leading to an increased production of platelets. The chemical, pharmacological, and therapeutic use aspects of lusutrombopag and avatrombopag are described in the new approvals section below.

### 2.2. Spleen Tyrosine Kinase (SYK)

Spleen tyrosine kinase (SYK) is a cytosolic protein tyrosine kinase which was discovered in 1990 [28]. The human SYK gene encodes a 635-amino acid polypeptide with an estimated molecular weight of 72,000 Da. The human kinase is expressed primarily in hematopoietic cells including B-cells, monocytes, macrophages, neutrophils, and mast cells. SYK affects cellular proliferation, differentiation, survival, and immune regulation via IgG Fc-receptor signaling. It is also linked to B-cell receptor signaling and auto-antibody production [29,30]. Thus, SYK inhibitors are being developed to treat allergic disorders as well as antibody-mediated autoimmune diseases such as allergic rhinitis, rheumatoid arthritis, asthma, cancer, diabetes type I, and immune thrombocytopenia among others. In 2018, a small molecule inhibitor of SYK known as fostamatinib was approved for the treatment of immune thrombocytopenia. Fostamatinib is a prodrug that undergoes bioactivation to produce R406, a potent SYK inhibitor. R406 inhibits signal transduction of Fc-activating receptors and B-cell receptor and reduces antibody-mediated platelets destruction. The chemical, pharmacological, and therapeutic use aspects of the newly approved SYK inhibitor prodrug, fostamatinib, are described in the new approvals section below.

## 3. The New Approvals

### 3.1. Lusutrombopag

**Figure 2:**
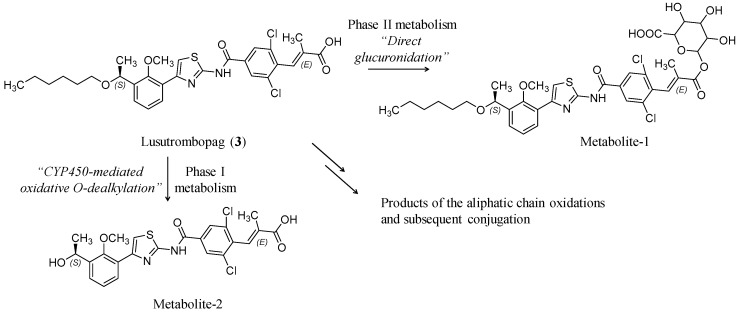
Structural representation of lusutrombopag (**3**). Presented also are two putative structures of two metabolites Metabolite-1 and Metabolite-2, which are the results of carboxylic acid O-glucuronidation (Phase II metabolism) or the side chain oxidative O-dealkylation (Phase I metabolism), respectively. No reports describe the pharmacological activity of the metabolites.

Lusutrombopag (**3**) (Mulpleta^®^, S-888711) (Figure 2) is a nonpeptide small molecule with the empirical formula of C_29_H_32_Cl_2_N_2_O_5_S and the molecular weight of 591.54. Chemically, it is (E)-3-[2,6-dichloro-4-[[4-[3-[(1S)-1-hexoxyethyl]-2-methoxyphenyl]-1,3-thiazol-2-yl]carbamoyl]phenyl]-2-methyl prop-2-enoic acid [31]. Specific structural features of lusutrombopag are described in Table 2.

Initially, the proliferative activity of lusutrombopag and its efficacy to induce megakaryocytic colony formation via human TPO-R was evaluated in cultured human c-Mpl-expressing Ba/F3 (Ba/F3-hMpl) cells and human bone marrow-derived CD34^+^ cells, respectively. Lusutrombopag resulted in a significant increase in Ba/F3-hMpl cells in a similar fashion to TPO and promoted colony-forming units-megakaryocyte and polyploid megakaryocytes in human CD34^+^ cells. In a genetically modified knock-in mouse model (TPOR-Ki/Shi), lusutrombopag significantly and dose-dependently increased circulating platelets during the time period of 21-day of recurrent oral administration. On day 22, the histopathological examination of the TPOR-Ki/Shi mice also indicated a substantial increase in megakaryocytes in the bone marrow [32].

Lusutrombopag was approved by FDA in July 2018 for the treatment of thrombocytopenia in adult patients with chronic liver disease who are scheduled to undergo a procedure. It was developed by Shionogi and was earlier approved in Japan in September 2015 for the improvement of chronic liver disease-associated thrombocytopenia in patients scheduled to undergo elective invasive procedures [33]. The recommended dose of lusutrombopag is 3 mg once daily for 7 days with or without food. The dosing should start 8–14 days before the scheduled procedure and patients should undergo their procedure 2–8 days following the last dose [34,35,36]. The increase in platelet count correlates with the AUC over the dose range of 0.25–4 mg of lusutrombopag in thrombocytopenic patients with chronic liver disease. With the recommended dosage (3 mg/day for 7 days), the mean maximum platelet count in patients without platelet transfusion is 86.9 × 10^3^/µL, and the median time to reach the maximum platelet count is 12 days (range: 5 to 35 days) [33,34].

The drug extensively binds to plasma protein (>99.9%), and thus, hemodialysis is not anticipated to enhance its elimination. Lusutrombopag’s elimination half-life is ~27 h. Furthermore, it is largely metabolized by CYP4 enzymes, including CYP4A11 [33,34]. Excretion in feces occurs for 83% of the dose with 16% excreted as unchanged drug. Excretion in urine accounts for ~1%. The major metabolic pathway for lusutrombopag appears to be ω- and β-oxidation and glucuronidation (Figure 2). The level of the reported effects of the two systems, kidney and liver, on the pharmacokinetic profile of lusutrombopag suggested that no adjustment is required in patients with mild-to-moderate renal and/or liver impairments. According to in vitro data, lusutrombopag is a substrate of breast cancer resistance protein (BCRP) and P-glycoprotein/ABCB1. Lusutrombopag does not induce or inhibit cytochrome CYP450 3A activity at therepaeutic doses [33,34]. 

Notably, differences in age, race, and/or ethnicity do not appear to lead to clinically significant differences in the pharmacokinetic aspects of lusutrombopag. Furthermore, although exposure to lusutrombopag tends to decrease with increasing body weight, yet this decrease is not considered clinically relevant. Moreover, a population pharmacokinetic analysis did not find a clinically significant effect of mild and moderate renal dysfunction on the pharmacokinetics of lusutrombopag. Data in patients with severe renal impairment are limited. Likewise, no clinically significant changes in the pharmacokinetic aspects of lusutrombopag were observed in case of mild to moderate liver dysfunction. Nevertheless, the mean observed C_max_ and AUC diminished by 20–30% in patients with severe liver dysfunction in relative to those with mild and moderate liver disease [37].

The most frequent side effect of lusutrombopag is headache with a frequency of 5%. Lusutrombopag is also associated with a limited risk of thrombotic and thromboembolic complications, with portal vein thrombosis being the most frequent (1%; 2 cases out of 171 participants in 3 clinical trials) [33,34]. Interestingly, detailed toxicological studies were conducted in mice and rats and revealed no carcinogenicity to mice at oral doses up to 20 mg/kg/day in males and females or to rats at oral doses up to 20 mg/kg/day in males and 2 mg/kg/day in females [33,34]. Lusutrombopag is not genotoxic based on an in vitro bacterial reverse mutation (AMES) assay and an in vivo micronucleus assay with mouse bone marrow cells. Likewise, lusutrombopag does not affect fertility in male and female rats at oral doses up to 100 mg/kg/day as it was demonstrated in a fertility and early embryonic development test [33,34]. Important to mention also is that lusutrombopag does not prolong the QT interval to a clinically significant extent using a dose of 24 mg which suggests the lack of significant cardiac toxicity at doses higher than the therapeutic ones [33,34].

The clinical efficacy of lusutrombopag in treating thrombocytopenia in patients with chronic liver disease who are scheduled for a procedure was examined in two randomized, double-blind, placebo-controlled trials (L-PLUS 1 (*n* = 97) [38] and L-PLUS 2 (*n* = 215; NCT02389621) [39]). In the two trials, responders were defined as patients who had a platelet count of ≥50 × 10^3^/µL with an increase of ≥20 × 10^3^/µL from baseline. Specifically, in L-PLUS 1, the major efficacy outcome was the percentage of patients who needed no platelet transfusion before the primary invasive procedure. In L-PLUS 2, the major efficacy outcome was the percentage of patients who did not require platelet transfusion before the primary invasive procedure and did not require rescue therapy for bleeding from the time of randomization through 7 days after the primary invasive procedure. In L-PLUS 1, the proportion of participants who met the major efficacy outcome was 78% (vs 13% in the placebo group; *p* < 0.0001). About 76% responded to the therapy during the study vs. 6% in the placebo group (*p* < 0.0001). In L-PLUS 2, the proportion of participants who met the major efficacy outcome was 65% (vs. 29% in the placebo group; *p* < 0.0001). About 65% responded to the therapy during the study vs. 13% in the placebo group (*p* < 0.0001) [38,39].

In another study, eight patients with hepatocellular carcinoma and a platelet count of <50 × 10^3^/μL, before initial and repeat radiofrequency ablation at the time of recurrence, orally received lusutrombopag (3 mg/day for 7 days). The results indicated that the platelet count increased to 103.1 ± 22. 8 ×10^3^/μL and to 110.7 ± 17.8 ×10^3^/μL 14 days after the first treatment and 14 days after the repeated use, respectively. None of the patients needed platelet transfusion or developed serious adverse events of thrombosis, bleeding, fever, or rash [40]. Likewise, a successful case of avoidance of platelet transfusion with re-administration of lusutrombopag before radiofrequency ablation in patient diagnosed with hepatitis C, liver cirrhosis, and hepatocellular carcinoma was reported in Japan [41]. Another case concluded that repeated administration of lusutrombopag (3 mg/day for 7 days) is effective strategy for patients with thrombocytopenia to avoid platelet transfusion in patients with chronic liver disease who undergo two or more planned invasive procedures including invasive hepatocellular carcinoma treatment [42].

Furthermore, a reported case of a patient with compensated liver cirrhosis due to hepatitis C virus indicated that lusutrombopag not only promotes the proliferation and differentiation of bone marrow progenitor cells into megakaryocytes, and subsequently, increase platelet count, but also promotes the proliferation and differentiation of hematopoietic progenitors to subsequently increase the blood leukocyte and erythrocyte counts [43]. In another report, lusutrombopag appeared to be used successfully to treat thrombocytopenia in one patient, that is associated with cirrhosis attributed to hepatitis C virus and alcohol consumption, before partial splenic embolization. Yet, another patient developed disseminated intravascular coagulation [44]. Moreover, the effectiveness of lusutrombopag to treat thrombocytopenia in cirrhotic patients with low platelet counts before invasive procedures was evaluated in 25 patients. In all patients, platelet counts significantly increased from 41 ± 11 × 10^3^/µL to 82 ± 26 × 10^3^/µL (*p* < 0.01). Only 16% of the patients required platelet transfusion prior to the invasive procedures as compared to 43–66% of the cirrhotic patients without lusutrombopag (16% vs. 54%, *p* = 0.001). Interestingly, hemorrhagic complications were not observed. Portal thrombosis happened in one patient who had a history of thrombosis and was effectively treated by thrombolysis therapy. Collectively, these results suggest that the standard regimen of lusutrombopag (3 mg/day for 7 days) is a safe and effective drug for thrombocytopenia in cirrhotic patients and can diminish the need for frequent platelet transfusions [45]. Along these lines, another case report indicated that lusutrombopag is effective pretreatment for liver biopsy following liver transplantation in a pediatric patient and suggested that additional studies are needed to expand its clinical indications [46].

To document the effects of lusutrombopag beyond platelet count, a retrospective, multicenter study was conducted at four locations in Japan in which 50 thrombocytopenic patients with chronic liver disease were evaluated for general changes following treatment with the standard regimen of lusutrombopag i.e., 3 mg/day for 7 days. Based on the reported definition of response, the numbers of responders and non-responders were 40 patients (80%) and 10 patients (20%), respectively. Splenic volume (*p* < 0.001) and body weight (*p* = 0.044) were lower in responders than in non-responders. Hemoglobin level (*p* = 0.026) and white blood cell count (*p* = 0.02) were higher in the responder group in relative to the non-responder group [47].

### 3.2. Avatrombopag 

**Figure 3:**
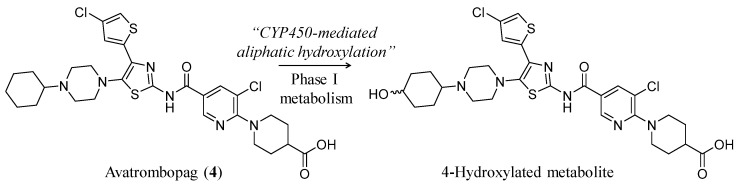
Structural representation of avatrombopag (**4**) which has multicyclic chemical structure with a terminal carboxylic group and a central domain of substituted 1,3-thiazol-2-yl carbamoyl aryl moiety. Presented also is the reported CYP450-phase I metabolism which results in the formation of 4-hydroxylted metabolite(s). No reports describe the pharmacological activity of the metabolites.

Avatrombopag (**4**) (Doptelet^®^, AKR-501 (YM477)) (Figure 3) is another nonpeptide small molecule agonist of TPO-R. It has a multicyclic chemical structure with the empirical formula of C_29_H_34_Cl_2_N_6_O_3_S_2_ and the molecular weight of 649.65. Similar to lusutrombopag, it has a terminal carboxylic group and a central domain of substituted 1,3-thiazol-2-yl carbamoyl aryl moiety. Its chemical name is 1-[3-chloro-5-[[4-(4-chlorothiophen-2-yl)-5-(4-cyclohexylpiperazin-1-yl)-1,3-thiazol-2-yl]carbamoyl]pyridin-2-yl]piperidine-4-carboxylic acid [48]. Specific structural features of avatrombopag are summarized in Table 2.

Avatrombopag was developed by AkaRx (parent organization is Dova Pharmaceuticals) and was approved by FDA in May 2018. It is indicated for the treatment of thrombocytopenia in adult patients with chronic liver disease who are scheduled to undergo a procedure [49,50,51,52]. In the early stage of development, it was reported that avatrombopag specifically targeted the TPO-R and stimulated megakaryocytopoiesis in a similar fashion to recombinant human TPO. Avatrombopag exhibited efficacy only in chimpanzees and humans with high species specificity. Oral administration of avatrombopag on daily basis dose-dependently increased the number of human platelets in mice model transplanted with human fetal liver CD34^+^ cells, with a significant increase achieved at doses of ≥1 mg/kg [48]. Furthermore, avatrombopag did not inhibit TPO binding to its receptor. This result indicated that avatrombopag and TPO may act simultaneously on the receptor and exhibit additive effect on megakaryocytopoiesis [53]. In contrast to TPO which lowers the threshold for platelet activation, avatrombopag did not increase the number of circulating activated platelets as measured by platelet surface P-selectin and activated glycoprotein 2b/3a or the platelet reactivity to low or high concentrations of thrombin receptor-activating peptide and ADP in randomized, double-blind, placebo-controlled, parallel-group study of chronic liver disease patients with thrombocytopenia [54].

Given its highly lipophilic nature, avatrombopag is prepared as maleate salt with a ratio of 1:1 to increase its aqueous solubility [55]. The drug is provided as an immediate-release tablet containing 20 mg avatrombopag. The recommended daily dose of avatrombopag is based on the patient’s platelet count before the scheduled procedure with a duration of treatment for 5 days. The drug should be taken orally once daily (2 or 3 tablets) for 5 consecutive days with food. The dosing should start 10–13 days before the scheduled procedure and patients should undergo the scheduled procedure 5 to 8 days following the last dose [49,50]. The drug extensively binds to plasma protein (>96%). Platelet count increases within 3–5 days with the peak of platelet count increase is observed after 10–13 days. Platelet count steadily declines within 7 days of the procedure and platelet count returns to baseline in about 35 days. Elimination half-life of the drug is ~19 h. The most frequent side effects of ≥3% are pyrexia, nausea, fatigue, abdominal pain, headache, and peripheral edema [52,55,56,57]. Similar to drugs with the same mechanism of action, avatrombopag is associated with a slight risk of thrombotic and thromboembolic complications, particularly portal vein thrombosis (1 case out of 430 participants in clinical trials ADAPT-1 (NCT01972529) and ADAPT-2 (NCT01976104)) [58].

Avatrombopag is a substrate of CYP2C9 and CYP3A, which metabolize the drug to the corresponding 4-hydroxylated products with the former enzyme playing more predominant role (Figure 3) [52,55,56,57]. Given this metabolic profile, drug-drug interactions between avatrombopag and rifampin, enzalutamide, fluconazole, and mifepristone were reported. In a pharmacokinetic study of 16 healthy volunteers, rifampin (600 mg given daily on days 1 to 16) decreased the AUC and the half-life of avatrombopag (20 mg single dose given on day 7) by 43% and 52%, respectively. Return to the baseline platelet count occurred 7 days after avatrombopag administration when combined with rifampin, while return to baseline platelet count did not occur until 28 days after avatrombopag administration when it was given alone. Nevertheless, the maximum platelet count achieved was unchanged by rifampin coadministration [52,55,56,57]. The mechanism of this interaction is thought to be due to rifampin-mediated induction of CYP2C9 and CYP3A4. Enzalutamide is also a dual inducer of CYP2C9 and CYP3A4 and is expected to decrease the patient exposure to avatrombopag to a similar degree. Likewise, in a pharmacokinetic study of 16 healthy volunteers, fluconazole (400 mg given daily on days 1 to 16) increased the AUC and the maximum serum concentration of avatrombopag (20 mg single dose administered on day 7) by 2.16-fold and 1.17-fold, respectively. Pharmacodynamic effects of avatrombopag; the mean profile of platelet count over time and the change in the maximum platelet count, increased significantly in the presence of fluconazole. The mechanism of this interaction is thought to be due to fluconazole-mediated inhibition of CYP2C9 and CYP3A4. Likewise, mifepristone, which is also a dual inhibitor of CYP2C9 and CYP3A4, increased the patient exposure to avatrombopag to a similar degree [52,55,56,57]. Importantly, age (18–84 years), body weight, sex, race, hepatic dysfunction, or mild-to-moderate kidney dysfunction does not appear to exhibit clinically significant effects on the pharmacokinetic aspects of avatrombopag. However, the effect of severe kidney impairment on avatrombopag pharmacokinetics is unknown [52,55,56,57].

In extensive carcinogenicity studies, avatrombopag was orally administered at three doses (20, 60, and 160 mg/kg/day) in mice and three doses (20, 50, and 160 mg/kg/day) in rats. Avatrombopag induced a statistically significant increase in gastric carcinoids at 160 mg/kg in female rats. The carcinoids in rodents were thought to be due to prolonged hypergastrinemia which is claimed to be of a low risk or relevance to humans. Nevertheless, avatrombopag is not mutagenic and does not affect fertility or early embryonic development in male rats or female rats as demonstrated in a host of in vitro and in vivo tests following high dose exposures [49]. Furthermore, avatrombopag (40 mg and 60 mg) does not prolong the QT interval to a clinically relevant extent suggesting the lack of significant cardiac toxicity at doses higher than the therapeutic ones.

The clinical efficacy of avatrombopag in treating thrombocytopenia in patients with chronic liver disease who are scheduled to undergo a procedure was established in two multicenter, randomized, double-blind, placebo-controlled trials (ADAPT-1 (*n* = 231) and ADAPT-2 (*n* = 204)) [50,58]. In each study and based on the platelet count, patients were assigned to the Low Baseline Platelet Count Cohort (˂40 × 10^3^ platelets/µL), who were treated with 60 mg drug or placebo once daily for 5 days, or the High Baseline Platelet Count Cohort (≥40 to ˂50 × 10^3^ platelets/µL), who received 40 mg drug or placebo once daily for 5 days. The major efficacy outcome was the percentage of patients who needed no platelet transfusion or any rescue procedure for bleeding from the time of randomization and up to 7 days following an elective procedure. Additional secondary efficacy outcomes were the percentage of patients who had platelet counts of ≥50 × 10^3^ platelets/µL on the day of procedure as well as the change in platelet count from the baseline to the day of the procedure [50,58].

In ADAPT-1, about 66% in the Low Baseline Platelet Count Cohort responded to the therapy during the study vs. 23% in the placebo group (*p* < 0.0001). Likewise, in ADAPT-2, approximately 69% in the Low Baseline Platelet Count Cohort responded to the therapy during the study vs. 35% in the placebo group (*p* < 0.0001) [50,58]. In ADAPT-1, about 88% in the High Baseline Platelet Count Cohort responded to the therapy during the study vs. 38% in the placebo group (*p* < 0.0001). Likewise, in ADAPT-2, about 88% in the High Baseline Platelet Count Cohort responded to the therapy during the study vs. 33% in the placebo group (*p* < 0.0001) [50,58]. Furthermore, the two trials demonstrated a higher percentage of participants who achieved the platelet count of ≥50 × 10^3^ platelets/µL on the day of the procedure in avatrombopag groups versus placebo groups for the two cohorts. Moreover, the trials showed a greater mean change in platelet counts from the baseline to the procedure day in the two avatrombopag groups for the two cohorts. A measured increase in platelet counts was observed in avatrombopag groups over time starting on day 4. This increase reached the peak on day 10–13, declined 7 days after the procedure, and then returned back to near baseline by day 35 [50,58].

Lastly, considering the two newly FDA-approved TPO-R agonists; lusutrombopag and avatrombopag, the effectiveness and the safety of treatment in pediatric patients have not been thoroughly established. Furthermore, differences in responses between the younger and the elderly patients have not been identified. In case of overdose, there is no antidote, and patients should be treated from thrombotic/thromboembolic complications following the standards of care. Hemodialysis is not effective in treating overdose due to the extensive binding of the two drugs to plasma proteins [34,49]. Considering results from animal studies, female patients who are planning to become pregnant should be counseled that the use of lusutrombopag is possible if the benefit justifies the potential risk to the fetus [34]. In addition, breastfeeding is to be avoided during the treatment with lusutrombopag and for at least 28 days following the last dose [34]. Likewise, avatrombopag may also adversely affect the fetus when administered to pregnant patients and breastfeeding is not endorsed during the treatment course and for at least 14 days following the last dose [49].

### 3.3. Fostamatinib

**Figure 4:**
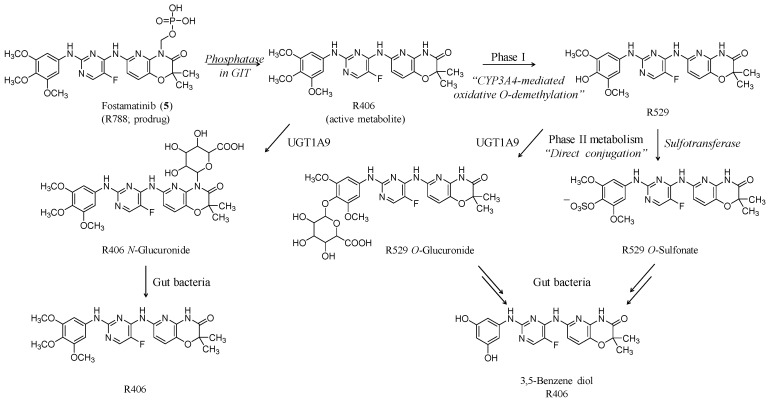
Structural representation of fostamatinib (**5**) which has a central domain of fluorinated amino-pyrimidine that is substituted from one side by trimethoxy aniline and from the other side with dimethyl-pyrido-oxazinone. The latter is the site at which the prodrug moiety is installed. Fostamatinib is metabolized in the gut by alkaline phosphatase to R406 which is the active metabolite. R406 is extensively metabolized, primarily via oxidative O-demethylation (phase I metabolism) and N- or O-glucuronidation (phase II metabolism). No reports describe the pharmacological activity of other metabolites.

Fostamatinib (**5**) (Tavalisse^®^, R-788) (Figure 4) is the third nonpeptide small molecule drug that was approved for thrombocyopenia by FDA last year. It has the empirical formula of C_23_H_26_FN_6_O_9_P and the molecular weight of 580.15. Structurally, fostamatinib has a central domain of fluorinated amino-pyrimidine that is substituted from one side by trimethoxy aniline moiety “left-hand domain” and from the other side with dimethyl-pyrido-oxazinone moiety “right-hand domain”. The latter is the site at which the methylene phosphate prodrug moiety is installed. Its chemical name is [6-[[5-fluoro-2-(3,4,5-trimethoxyanilino)pyrimidin-4-yl]amino]-2,2-dimethyl-3-oxopyrido[3,2-b][1,4]oxazin-4-yl]methyl dihydrogen phosphate. The drug is used orally in the form of hexahydrate disodium salt to enhance its oral bioavailability [59,60]. Specific structural features of fostamatinib are summarized in Table 2 above.

Fostamatinib was approved by FDA in April 2018 to treat chronic and refractory immune thrombocytopenia in patients who have had insufficient response to previous treatment including corticosteroids, immunoglobulins, splenectomy, and/or a TPO-R agonist [59,60]. Fostamatinib is a prodrug that is biochemically transformed in the GIT to the active metabolite R406 [61], which inhibits SYK activity with an IC_50_ value of 41 nM. The active metabolite recognizes the ATP binding pocket, and thus, competitively blocks the ATP binding with a K_i_ value of 30 nM. It is 5- to 100-fold more selective to SYK over a panel of >90 other kinases. Functional in vitro studies with R406 indicated that it is a specific inhibitor of SYK-dependent FccR-mediated signaling in human mast cells, neutrophils, and macrophages [62,63]. It also blocks B-cell receptor-mediated activation of B lymphocytes. In murine models, administration of 25 or 40 mg/kg of fostamatinib prevented the development of thrombocytopenia in mice injected with antibody targeting integrin aIIb as well as the development of anti-red blood cell antibody-mediated anemia [64]. Other SYK inhibitors including entospletinib and cerdulatinib are being developed for hematological malignancies and do demonstrate different selectivity in comparison to fostamatinib [29].

Fostamatinib was developed by Rigel Pharmaceuticals and is available as 100 mg or 150 mg film-coated tablets. The drug is orally initiated at a dose of 100 mg taken twice daily. If the platelet count does not increase to at least 50 × 10^3^ platelets/µL after a month, the dose is increased to 150 mg twice daily [59,60]. The drug may be consumed with or without food and it should be stopped after 12 weeks of treatment if the count of platelets does not rise to a level enough to avoid bleeding [59,60]. The most frequent adverse reactions of ≥5% frequency than placebo are diarrhea (31%), hypertension (28%), nausea (19%), dizziness (11%), respiratory infection (11%), hepatotoxicity (increased aspartate aminotransferase and alanine aminotransferase; 11%), rash (9%), abdominal pain (6%), fatigue (6%), chest pain (6%), and neutropenia (6%) [65].

As indicated above, fostamatinib is metabolized in the gut by alkaline phosphatase to R406, which is the active metabolite. Oral bioavailability of R406 is 55% [66]. Median time to platelet count of ≥50 × 10^3^ platelets/µL is 15 days. The active metabolite volume of distribution is 256 ± 92 L/kg with protein binding potential of 98%. Elimination half-life of R406 is 15 ± 4.3 hrs. R406 is extensively metabolized, primarily via oxidation (CYP3A4, Phase I) and glucuronidation (UGT1A9; Phase II) (Figure 4). The resulting metabolites are eliminated in feces (~80%) and urine (~20%) [59,60,61]. Surprisingly, a significant pharmacological relationship was demonstrated between the time course of the blood pressure effect and changes in the plasma concentration of the active metabolite. Importantly, it was demonstrated that the blood pressure increase induced by fostamatinib administration can be effectively managed by drug withdrawal or co-dosing with known antihypertensive drugs including captopril, atenolol, and nifedipine. In fact, the blood pressure control can be achieved without compromising the therapeutic benefit of fostamatinib [67].

In addition to being a substrate to CYP3A4 and UGT1A9, fostamatinib also inhibits BCRP and P-glycoprotein/ABCB1. This pharmacokinetic profile leads to several drug-drug interactions. The concurrent use of fostamatinib with a strong CYP3A4 inducer reduces exposure to the active metabolite, and thus, it is not recommended. CYP3A4 inducers include phenytoin, fosphenytoin, carbamazepine, phenobarbital, primidone, apalutamide, enzalutamide, mitotane, lumacaftor, and rifampin. In a pharmacokinetic study of 15 healthy volunteers, rifampin (600 mg given daily for 8 days) co-administered with fostamatinib (150 mg single dose given on day 6), decreased the AUC and the maximum serum concentration of R406 by 75% and 59%, respectively [68]. Furthermore, P-glycoprotein/ABCB1 inhibitors such as fostamatinib may increase the serum concentration of P-glycoprotein/ABCB1 substrates including betrixaban, edoxaban, dabigatran etexilate, doxorubicin, ranolazine, bosentan, and colchicine among others. This is because fostamatinib may increase the systemic absorption and decrease the renal and/or the biliary elimination of P-glycoprotein/ABCB1 substrates. P-glycoprotein inhibitors may also increase the distribution of P-glycoprotein substrates to specific tissues or organs where P-glycoprotein is abundant [60,69]. Along these lines, fostamatinib exhibited drug-drug interactions when co-administered with oral contraceptive (ethinyl estradiol/ levonorgestrel), rosuvastatin, or simvastatin with the AUC of statins is almost doubled. Fostamatinib did not demonstrate a clinically significant drug-drug interaction with warfarin [70]. Likewise, fostamatinib was not found to have clinically meaningful effect on the pharmacokinetics of pioglitazone and other CYP2C8 substrates [71].

In 2-year carcinogenicity studies, fostamatinib was neither carcinogenic in mice (oral gavage at doses up to 500/250 mg/kg/day) nor carcinogenic in rats (oral gavage at 45 mg/kg/day). Neither fostamatinib nor R406 was mutagenic in an in vitro AMES assay. They were not clastogenic in an in vitro human lymphocyte chromosomal aberration assay or an in vivo mouse bone marrow micronucleus assay [60]. All mating, sperm number and motility, and organ weight measures in male rats were not affected by doses as high as 40 mg/kg/day in a fertility study with oral fostamatinib. All mating and fertility measures in female rats were also not affected by doses as high as 11 mg/kg/day, but a slight decrease in pregnancy rates was seen at 25 mg/kg/day [60].

At about 300 mg, the drug does not prolong the QT interval to a clinically significant extent suggesting the lack of significant cardiac toxicity at doses higher than the therapeutic ones [60]. Population pharmacokinetics analyses indicated that the exposure to the drug is not affected by sex, age, race, or ethnicity [59,60]. Furthermore, the drug’s pharmacokinetics is not modified in patients with renal or hepatic impairment [72]. As with the new TPO-R agonists above, the effectiveness and safety of fostamatinib in pediatric patients have not been established. In case of overdose, there is no antidote, and patients should be treated from thrombotic/thromboembolic complications following the standards of care [60]. Based on animal studies, fostamatinib may cause fetal consequences when used by pregnant patients. Thus, female patients are to be advised to use contraception during the treatment period and for at least 1 month following the last dose. Breastfeeding is also to be avoided during the treatment period with fostamatinib and for at least 30 days following the last dose [60].

The efficacy and safety of fostamatinib in the treatment of thrombocytopenia were evaluated in two placebo-controlled studies (FIT-1 (*n* = 76; NCT02076399) and FIT-2 (*n* = 74; NCT02076412) [65]), and in an open-label extension study (FIT-3 (*n* = 123; NCT 02077192) [60]. Following the administration of 100 mg fostamatinib or placebo treatment two times a day, the efficacy of fostamatinib was decided based upon getting a steady platelet count of at least 50 × 10^3^/µL on at least 4 of the 6 visits between weeks 14 and 24. Considering the platelet count and tolerability, the dose was escalated to 150 mg twice daily in 88% of patients at week 4 or after. Patients who did not achieve the targeted platelet count after 12 weeks, and those who finished the 24-week double blind study, were qualified to enroll in the open-label extension study (FIT-3). Steady response in FIT-3 was prospectively defined as no 2 visits, at least 4 weeks apart, with a platelet count of <50 × 10^3^/µL, with no intervening visit with a platelet count of at least 50 × 10^3^/µL, within 12 weeks after the initial achievement of the desired platelet count. In the FIT-1 and FIT-2 studies, 17% of the patients achieved a stable response to fostamatinib. In FIT-3, 23% met the definition for steady response. Among the patients who achieved steady response in the above trials, 18 patients maintained the platelet count of at least 50 × 10^3^/µL for 12 months or longer.

## 4. Conclusions and Future Directions

In one hand, lusutrombopag and avatrombopag are new FDA-approved TPO-R nonpeptide agonists that are highly effective in raising the platelet count in thrombocytopenic patients with chronic liver disease. When compared with other agonists, they continue to have minimal adverse effects, are not associated with serious drug-drug interactions, and require no adjustment in renal and/or liver impairment (See Supplementary Table S1). They are also not associated with clinically significant hepatotoxicity. In the other hand, fostamatinib presents a new class of therapy with fundamentally novel mechanism of action that is different from all the other available therapies. Its active form is an inhibitor of SYK, and it is the only currently approved therapy for the treatment of chronic immune thrombocytopenia that does not respond to previous treatment including immunoglobulins, corticosteroids, splenectomy, and/or a TPO-R agonist. Furthermore, it is well known that the need for the weekly injection of romiplostim as well as the dietary limitations with eltrombopag complicate their use. In fact, eltrombopag should be taken on an empty stomach with no food 2 h before and after the drug consumption, and no calcium- or iron-containing products or supplements for 4 h before and after the drug consumption. In contrast, all the newly FDA-approved drugs are orally effective, and their absorption profiles are less impacted by food consumption with no anticipated effect presented by supplements containing polyvalent cations [73].

A recent systemic review and meta-analysis of thirteen randomized controlled trials with 1202 patients was conducted to establish a clinically meaningful ranking of the safety and efficacy of treatments for adults who did not respond to first-line treatment for immune thrombocytopenia or who relapsed following response requiring further treatment [74]. The report included the use of romiplostim, avatrombopag, eltrombopag, fostamatinib, and rituximab. The report concluded that, in adults of ≥18 years, romiplostim seems to be the most effective treatment in terms of overall response of achieving platelet count of ≥50 × 10^3^/µL at the end of treatment without the need for rescue therapy, followed by avatrombopag, eltrombopag, fostamatinib, and rituximab. Moreover, avatrombopag produced more satisfactory outcomes than romiplostim, rituximab, and eltrombopag in terms of early response of achieving a platelet count of ≥50 × 10^3^/µL at the second week after initiation of treatment [74]. In a different report, the relative potency of the TPO-R agonists avatrombopag, eltrombopag, and romiplostim in a patient with chronic immune thrombocytopenia due to systemic lupus erythematosus was reported [75]. In one hand, a daily 20 mg dose of avatrombopag achieved a peak platelet count that is 3–5 times higher than that achieved with daily 75 mg doses of eltrombopag. In the other hand, romiplostim at the maximal doses produced a peak platelet count that is 8–10 times higher than that achieved with the maximal doses of eltrombopag. Accordingly, this report further supports the preceding report related to the relative efficacy of different TPO-R agonists.

Another recent meta-analysis study reported on the relationship between TPO-R agonists (eltrombopag, lusutrombopag, and avatrombopag) and the risk of portal vein thrombosis as well as arterial and venous thrombo-embolic events in patients with chronic liver disease and thrombocytopenia [76]. The authors looked at four studies including 1953 patients and found no significant difference for the incidence of portal vein thrombosis in patients treated with the agonists compared with the placebo (*p* = 0.055), even in the case of surgeries. Yet, a significant association was detected only in patients treated with eltrombopag (*p* = 0.03) [76]. Further analysis by the authors included three studies of 514 patients suffering from thrombocytopenia and chronic liver disease and undergoing an elective invasive procedure concluded no significant difference for the incidence of portal vein thrombosis between the agonists group and the placebo group (*p* = 0.212). A third analysis evaluated the incidence of arterial and venous thrombo-embolic events in two trials in which 1727 patients were randomized to eltrombopag or placebo. A significant difference was observed for the incidence of these events in chronic liver disease patients treated with eltrombopag in relative to those treated with placebo (*p* = 0.003) [76]. Nevertheless, the increased thrombo-embolic events in the eltrombopag group can be because of the screening and exclusion criteria of L-PLUS (lusutrombopag) and ADAPT (avatrombopag) trials given their late development [77]. Considering long-term adverse effects, a single-center, long-term follow-up study found that TPO-R agonists (romiplostim, eltrombopag, and/or avatrombopag) induce myelofibrosis grades 2/3 in about 20% of patients with immune thrombocytopenia, particularly with more than 2 years of treatment. Therefore, a follow-up with bone marrow biopsies is recommended in order to allow prompt discontinuation [78].

Lastly, given their efficacy and safety profiles, the new drugs will continue to be evaluated in other cases of thrombocytopenia. For example, the clinical trial for using avatrombopag in the treatment of chemotherapy-induced thrombocytopenia in adults with active non-hematological cancers is ongoing (NCT03471078).

## Figures and Tables

**Table 1:** Drugs associated with thrombocytopenia.

Types	Drugs
Drug-induced immune thrombocytopenia	Abciximab, Acetaminophen, Amiodarone, Carbamazepine, Ceftriaxone, Daptomycin, Eptifibatide, Ethambutol, Furosemide, Haloperidol, Heparin, Ibuprofen, Irinotecan, Levofloxacin, Mirtazapine, Naproxen, Oxaliplatin, Piperacillin, Phenytoin, Quinidine, Ranitidine, Rifampin, Simvastatin, Sulfonamides including Trimethoprim-sulfamethoxazole, Suramin, Tirofiban, Vancomycin
Dose-dependent bone marrow suppression	Daptomycin, Gold compounds, Linezolid, Valproic acid

**Table 2:** Properties of the new drugs.

Predicted Properties	Lusutrombopag (3)	Avatrombopag (4)	Fostamatinib (5)
LogP *	8.08	7.09	3.24
LogS *	−8.805	−8.347	−5.03
pKa *	3.778	4.501 and 8.127	1.46 and 2.71
Polar surface area *	97.22	100.84	185.13
Refractivity *	158.17	170.04	136.52
H-Bond acceptor	6	8	13
H-Bond donor	2	2	4
Rotatable bonds	13	7	10
Number of rings	3	6	4

* Calculated by ChemDraw; P is partition coefficient and S is aqueous solubility; Unit for polar surface area is Å^2^ and for refractivity is m^3^·mol^−1.^

## Supplementary Materials

Supplementary materials can be found at https://www.mdpi.com/1422-0067/20/12/3013/s1.

## Abbreviations

BCRP	Breast cancer resistance protein
HIT	Heparin-induced thrombocytopenia
JAK	Janus kinase
MAPK	Mitogen-activated protein kinase
PI3K	Phosphatidylinositol-3 kinase
STAT	Signal transducer and activator of transcription
SYK	Spleen tyrosine kinase
TPO-R	Thrombopoietin receptor
