# Supplementary material for: New Small Molecule Drugs for Thrombocytopenia: Chemical, Pharmacological, and Therapeutic Use Considerations
Source: Int J Mol Sci. 2019 Jun 20;20(12):3013. doi: 10.3390/ijms20123013 (PMC6628068; doi:10.3390/ijms20123013)
Supplement: Supplementary file 1 [file ijms-20-03013-s001.pdf]

New Small Molecule Drugs for Thrombocytopenia: Chemical, Pharmacological, and Therapeutic Use Considerations

Page Clemons Bankston[1] and Rami A. Al-Horani[1*]

[1]Division of Basic Pharmaceutical Sciences, College of Pharmacy, Xavier University of Louisiana, New Orleans, Louisiana 70125

***** Address for correspondence: Dr. Rami A. Al-Horani, 1 Drexel Drive, Suite 327, New Orleans, LA 70125-1089. Phone: (504) 520-7603, Fax: (504) 520-7954, Email: ralhoran@xula.edu

Table S1. FDA-approved TPO-R agonists and SYK inhibitor for immune thrombocytopenia.

Treatments	Romiplostim (Nplate®) (August 2008)	Eltrombopag (Promacta®) (November 2008)	Lusutrombopag (Mulpleta®) (July 2018)	Avatrombopag (Doptelet®) (May 2018)	Fostamatinib (Tavalisse®) (April 2018)
**Nature**	Fusion protein analog of TPO	Small molecule	Small molecule	Small molecule	Small molecule; prodrug
**Target**	TPO-R	TPO-R	TPO-R	TPO-R	SYK
**Administration**	Subcutaneous; 1 mcg/kg once weekly	Oral; 50 mg once daily	Oral; 3 mg once daily for 7 days	Oral; 40 or 60 mg once daily for 5 days depending on platelet count	Oral: Initial: 100 mg twice daily (may increase to 150 mg) for 12 weeks
**Approved indication(s)**	1) Treatment of thrombocytopenia in adult patients with chronic immune thrombocytopenia (ITP) who have had insufficient response to corticosteroids, immune globulin, or splenectomy 2) Treatment of thrombocytopenia in pediatric patients ≥1 year of age with ITP for ≥6 months who have had insufficient response to corticosteroids, immune globulin, or splenectomy	1) First-line treatment of severe aplastic anemia in patients ≥2 years 2) Treatment of severe aplastic anemia in patients who have had an insufficient response to immune-suppressive therapy 3) Treatment of thrombocytopenia in patients with chronic hepatitis C to allow the initiation and maintenance of interferon therapy 4) Treatment of thrombocytopenia in adult and pediatric patients ≥1 year of age with chronic immune thrombocytopenia who have had insufficient response to corticosteroids, immunoglobulins, or splenectomy	1) Treatment of thrombocytopenia in adult patients with chronic liver disease who are scheduled to undergo a procedure	1) Treatment of thrombocytopenia in adult patients with chronic liver disease who are scheduled to undergo a procedure	1) Treatment of thrombocytopenia in adults with chronic immune thrombo-cytopenia who have had an insufficient response to a previous treatment
**Onset of action**	4-9 days	7-14 days	Median 12 days	3-5 days	Median 15 days
**Elimination half-life**	Median 3.5 days (Platelet count returns to baseline in 28 days)	21-32 hours (Platelet count returns to baseline in 7-14 days)	27 hours	19 hours (Platelet count returns to baseline in 35 days)	15 (± 4.3) hours
**Drug interactions**	None is known	It is substrate of CYP3A4 and inhibitor of BCRP and P-glycoprotein	None is known	It is substrate of CYP2C9 (minor), CYP3A4 (minor), P-glycoprotein	It is substrate of CYP3A4 and UGT1A9 and inhibitor of BCRP and P-glycoprotein
**Frequent side effects**	Arthralgia, dizziness, insomnia, myalgia, pain in extremity, abdominal pain, shoulder pain, dyspepsia, and paresthesia	Nausea, vomiting, menorrhagia, myalgia, paresthesia, cataract	Headache	Pyrexia, abdominal pain, nausea, headache, fatigue, and edema peripheral	Diarrhea, hypertension, nausea, respiratory infection, dizziness, ALT/AST increased, rash, abdominal pain, fatigue, chest pain and neutropenia
**Others**	No adjustment in renal or liver impairment; Pregnancy risk C	Not studied in renal impairment; Half the dose is used in liver impairment; Pregnancy risk C	No adjustment in renal or liver impairment	No adjustment in renal or liver impairment	No adjustment in renal or liver impairment
